# The Influence of Edaphic Factors on DNA Damage and Repair in Wild Wheat *Triticum dicoccoides* Körn. (*Poaceae*, Triticeae)

**DOI:** 10.3390/ijms24076847

**Published:** 2023-04-06

**Authors:** Olga Raskina, Boris Shklyar, Eviatar Nevo

**Affiliations:** 1 Institute of Evolution, University of Haifa, Mt. Carmel, Haifa 3498838, Israel; 2 Bioimaging Unit, Faculty of Natural Sciences, University of Haifa, Mt. Carmel, Haifa 3498838, Israel

**Keywords:** DNA double-strand breaks, DNA repair, drought stress, gene expression, genome adaptation, R-loop, *Triticum dicoccoides*, wheat

## Abstract

A complex DNA repair network maintains genome integrity and genetic stability. In this study, the influence of edaphic factors on DNA damage and repair in wild wheat *Triticum dicoccoides* was addressed. Plants inhabiting two abutting microsites with dry terra rossa and humid basalt soils were studied. The relative expression level of seven genes involved in DNA repair pathways—*RAD51*, *BRCA1*, *LigIV*, *KU70*, *MLH1*, *MSH2*, and *MRE11*—was assessed using quantitative real-time PCR (qPCR). Immunolocalization of RAD51, LigIV, γH2AX, RNA Polymerase II, and DNA-RNA hybrid [S9.6] (R-loops) in somatic interphase nuclei and metaphase chromosomes was carried out in parallel. The results showed a lower expression level of genes involved in DNA repair and a higher number of DNA double-strand breaks (DSBs) in interphase nuclei in plants growing in terra rossa soil compared with plants in basalt soil. Further, the number of DSBs and R-loops in metaphase chromosomes was also greater in plants growing on terra rossa soil. Finally, RAD51 and LigIV foci on chromosomes indicate ongoing DSB repair during the M-phase via the Homologous Recombination and Non-Homologous End Joining pathways. Together, these results show the impact of edaphic factors on DNA damage and repair in the wheat genome adapted to contrasting environments.

## 1. Introduction

In plant evolution, allopolyploids exhibit the highest level of genetic complexity when two or more different genomes unify in one nucleus to create a new species [1,2]. Present-day allotetraploid wild emmer wheat *Triticum dicoccoides* Körn. (*Poaceae*, Triticeae) (genome AABB, 2n = 4x = 28) originated via hybridization between ancient diploid species of the genera *Triticum* and *Aegilops* [3,4,5] in the Fertile Crescent, probably about 360,000 years ago [6,7,8]. Hybridization between domesticated emmer tetraploid wheat and *Aegilops tauschii* Coss. (genome DD, 2n = 2x = 14) that occurred approximately 8500–9000 years ago gave rise to the common wheat *T. aestivum* L. (genome AABBDD, 2n = 6x = 42) [5]. Wild *T. dicoccoides* is an annual, self-pollinated grass occurring in the transition zone between Mediterranean and steppe phytogeographic provinces [9]. The extant wild relatives of cultivated wheat, particularly *T. dicoccoides*, have evolved in the Near East Fertile Crescent across multiple geographic ranges and ecological habitats, resulting in broad physical and biotic heterogeneity of natural populations and high adaptive genetic diversity. Wild *Aegilops/Triticum* species are broadly adaptive to multiple diseases, pests, and abiotic stresses, and they are important natural genetic resources for cultivated wheat improvement [9,10].

The adaptability of the genome to changing environments, especially with sharp climatic fluctuations, underlies the existence and evolution of species. Genetic and epigenetic genomic changes are accompanied by significant alterations in the complex nuclear repetitive DNA fraction and karyotype architecture. The current intraspecific polymorphism of repetitive DNA is closely related to ongoing chromosomal rearrangements, which typically result from erroneous DNA repair and recombination [11,12,13]. In particular, the generation of R-loops at transcriptionally active sites, a naturally occurring process in eukaryotic cells, is one of the potential sources of chromosomal aberrations. During transcription, the nascent RNA binds the template DNA strand, generating an RNA-DNA hybrid structure that displaces the non-template ssDNA. This three-stranded nucleic acid assembly is known as the R-loop in bacteria and eukaryotes, including plants [14,15,16,17]. R-loops are tightly associated with numerous cellular processes, including regulation of gene expression, replication, telomere stability, and DNA repair. The dysregulation of R-loops causes replication fork stalling, hyper-recombination, DNA damage, and chromosomal rearrangements, which increase genome instability [14,18,19].

A complex network of different DNA repair mechanisms combats the deleterious consequences of DNA damage and ensures the stability and vital functions of eukaryotic genomes [20,21,22]. DNA Damage Response (DDR) is one of the key pathways contributing to genome integrity and promoting cell cycle control under biotic and abiotic stresses; the signaling mechanisms of the DDR are highly conserved among eukaryotes with certain variations, including in plants [23]. As in yeasts and mammals, in plants, two phosphatidylinositol 3 kinase-like (PI3K) protein kinases, Ataxia Telangiectasia Mutated (ATM) [24] and ATM and Rad3-related (ATR) [25], play key roles in DDR signaling. ATM and ATR recognize double-strand DNA breaks (DSBs) and single-strand DNA breaks (SSBs), respectively. DSBs activate ATM signaling through the MRN (MRE11, RAD50, NBS1) complex, and ATR is recruited to single-stranded DNA [23,26]. ATM and ATR mediate phosphorylation of the DNA damage-related histone H2AX, which is necessary for the recruitment of signaling and repair factors; γH2AX (gamma-H2AX phosphorylated) foci can be visualized by immunofluorescence (IF). There is a close correlation between γH2AX foci and DSB number loss and DSB repair, allowing the evaluation of DSB repair in individual cells [27,28]. Both ATM and ATR signaling activates the Suppressor Of Gamma 1 (SOG1) transcription factor, which controls the expression of genes involved in DNA repair, cell cycle regulation, and cell death control [29,30].

Activated by the DDR pathway, Non-Homologous End Joining (NHEJ) and Homologous recombination (HR) are the two main mechanisms of DSB break repair [23]. DSBs, which are continuously generated by external and internal factors during the cell cycle, are among the most dangerous aberrations. If not resolved, they will destabilize the genome and potentially lead to cell death [12,31]. In somatic cells, HR operates mainly during the S and G2 phases. Radiation-sensitive 51 (RAD51) and Breast Cancer 1 (BRCA1) are the key proteins of the HR pathway [23]. Canonical NHEJ (cNHEJ) and alternative NHEJ (aNHEJ) are the sub-pathways of NHEJ, operating during all stages of the cell cycle [21]. Heterodimer KU70/KU80 (KU70, X-ray cross-complementing 6; KU80, X-ray cross-complementing 5), the KU complex plays a central role in promoting NHEJ. DNA ligation terminates cNHEJ by mobilizing various proteins, including LigIV (Ligase 4; X-ray repair cross-complementing 4) [21,32].

The Mismatch Repair (MMR) system corrects DNA mispairs and loops caused by replication errors; it plays important roles in transcription-associated repair, meiosis, and recombination [33]. In eukaryotes, there are main homologs of the MutS and MutL prokaryotic MMR genes. In plants, there are six *MSH* genes and *MSH7* that are specific to plants [21,33]. The MSH heterodimers act in lesion recognition and binding activities, and the MLH heterodimers perform the repair.

In this study, the influence of edaphic factors on DNA damage and repair in *T. dicoccoides* was addressed. The study was carried out on native plants from two adjacent geologically and edaphically contrasting microsites, with basalt and terra rossa soil types, in the Tabigha (“Evolution Slope”) population (Israel) located north of the Lake of Galilee (Kinneret Lake) in Upper Galilee [10]. We evaluated the expression levels of seven genes encoding proteins involved in DNA repair, specifically, *RAD51* and *BRCA1* (HR pathway), *LigIV* and *KU70* (NHEJ pathway), *MLH1* and *MSH2* (MMR pathway), and *MRE11* (DDR pathway) through the application of real-time quantitative PCR (qPCR). In parallel, we elucidated the cytological aspects of DNA damage and repair processes in mitosis through the immunodetection of RAD51, LigIV, and γH2AX in interphase nuclei and condensed chromosomes. In addition, we applied immunodetection of DNA-RNA hybrid [S9.6] and RNA Polymerase II (RNAPII) to address the issue of whether DNA lesions occurring during M-phase could be associated with post-interphase R-loop formation.

Our data show that, in plants of the water-deficient Terra Rossa microsite, the number of DSBs in interphase nuclei increases significantly, and the expression of genes that play a key role in the DDR, NHEJ, and MMR pathways is lower compared with that in plants of the humid Basalt microsite. The mean contribution of HR to DSB repair in both microsites seems to be similar; however, the average values mask genotype-dependent differences in the level of gene expression. Immunodetection of RAD51 and LigIV on condensed chromosomes indicates that both DSB repair pathways, HR and NHEJ, operate during the M-phase; nevertheless, a significant proportion of DSBs, which are marked by γH2AX foci, remain unresolved in late mitosis. Furthermore, γH2AX-associated RNAPII and S9.6 foci are revealed on condensed chromosomes, suggesting post-interphase R-loop formation that is a potential source of chromosome aberrations, posing a threat to genome stability. Together, these results show the influence of edaphic factors on DNA damage and repair processes in the wheat genome in contrasting environments.

## 2. Results

The relative expression of *RAD51*, *BRCA1*, *MLH1*, *MSH2*, *LigIV*, *KU70*, and *MRE11* was assessed using quantitative real-time PCR. Immunodetection of RAD51, LigIV, γH2AX, RNAPII, and DNA-RNA hybrid [S9.6] was performed in somatic interphase nuclei and metaphase chromosomes.

### 2.1. The Level of Expression of Genes Encoding Proteins Involved in DNA Repair Pathways Depends on the Genotype and Differs between the Basalt and the Terra Rossa Microsites

**Figure 1:**
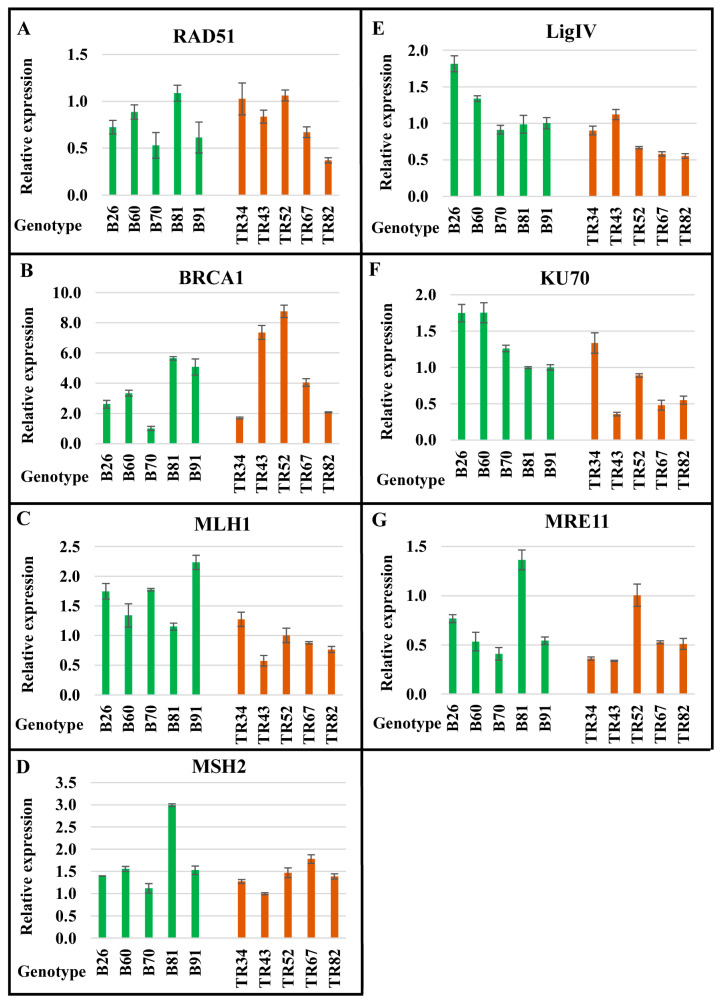
Real-time qPCR expression analysis of the genes involved in DNA repair pathways in individual plants from the Basalt (B) and the Terra Rossa (TR) microsites of the Tabigha population. The relative expression values of *RAD51* (**A**), *BRCA1* (**B**), *MLH1* (**C**), *MSH2* (**D**), *LigIV* (**E**), *KU70* (**F**), and *MRE11* (**G**) are represented. Standard deviation bars are shown.

Individual plants differ in the level of expression of each of the genes, both within and between the Basalt (B) and the Terra Rossa (TR) microsites of the Tabigha population. Thus, there was a threefold difference in the level of *RAD51* expression between the maximum in genotype B81 and the minimum in genotype TR82 (Figure 1). The expression level of *BRCA1* was almost ninefold higher in genotype TR52 than in genotype B70. The level of *MLH1* expression differed fourfold between the minimum and maximum in genotypes TR43 and B91, respectively. The level of *MSH2* expression differed by three times between the maximum in genotype B81 and the minimum in genotype TR43. More than a threefold difference was observed for *LigIV* between genotypes B26 and TR82. There was almost a fivefold difference for *KU70* between genotypes B26 and TR43. The expression level of *MRE11* was four times higher in genotype B81 compared with genotype TR43 (Figure 1).

**Figure 2:**
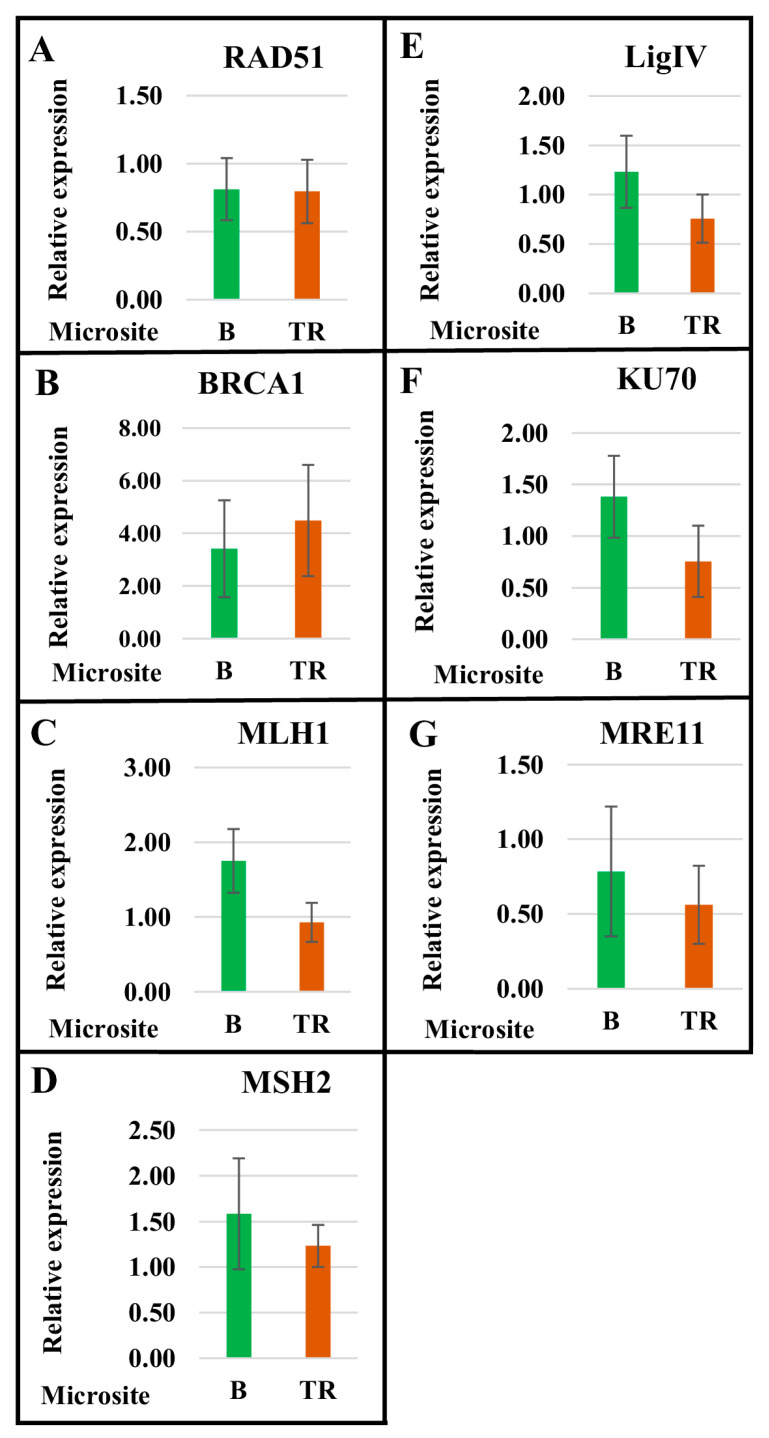
Real-time qPCR analysis of the mean expression of the genes involved in DNA repair pathways in plants from the Basalt (B) and the Terra Rossa (TR) microsites of the Tabigha population. The mean expression values of *RAD51* (**A**), *BRCA1* (**B**), *MLH1* (**C**), *MSH2* (**D**), *LigIV* (**E**), *KU70* (**F**), and *MRE11* (**G**) are represented for the B and TR microsites. Standard deviation bars are shown.

The mean expression values of genes involved in the MMR, NHEJ, and DDR pathways differed between the Basalt and Terra Rossa microsites. For *MLH1* and *MSH2* (MMR pathway), *LigIV* and *KU70* (NHEJ pathway), and *MRE11* (DDR pathway), the mean expression values were significantly higher in the Basalt microsite (Figure 2). In the case of HR, the mean expression level of *RAD51* was similar in the Basalt and Terra Rossa microsites, while *BRCA1* expression was higher in Terra Rossa, which contrasts with results for other genes (Figure 2).

Consequently, the average level of expression of the genes involved in the DDR (*MRE11*), NHEJ (*LigIV* and *KU70*), and MMR (*MLH1* and *MSH2*) pathways in plants of the dry Terra Rossa microsite is lower than that in plants of the humid Basalt microsite. However, the average expression level of *RAD51* is almost the same in both microsites, and the average expression level of *BRCA1* is higher in the Terra Rossa microsite (Figure 2).

In the Basalt microsite, a comparison of the expression of *RAD51*, *BRCA1*, *MRE11*, and *MSH2* between five plants (Figure 1) reveals the lowest level of expression in genotype B70; the highest level in genotype B81; and intermediate levels between B70 and B81 in relative values of gene expression among genotypes B26, B60, and B91. The patterns of *LigIV* and *KU70* expression are rather similar between plants of the Basalt microsite. In the Terra Rossa microsite, there is a similarity between *KU70* and *MLH1* relative expression patterns, where the highest and lowest expressions are found in genotypes TR34 and TR43, respectively. In genotypes TR52, TR67, and TR82, the expression values gradually decrease. The expression patterns of other genes vary significantly within the Terra Rossa microsite. Therefore, our data show that the intrapopulation variability in the expression levels of genes involved in the HR, NHEJ, MMR, and DDR pathways occurs in a genotype-dependent manner; moreover, they reveal differences between the moist Basalt and dry Terra Rossa microsites in terms of mean gene expression values.

### 2.2. DNA Repair Continues in the M-Phase with Genotype-Dependent Efficiency

**Figure 3:**
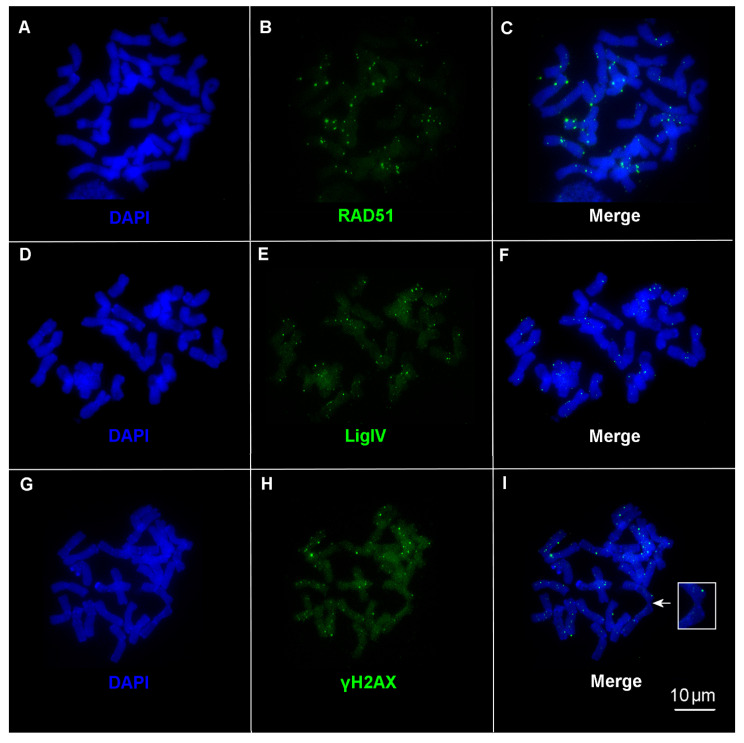
Immunodetection of RAD51, LigIV, and γH2AX on metaphase chromosomes. (**A**,**D**,**G**) Counterstaining with DAPI. Immunofluorescent localization of (**A**–**C**) anti-RAD51 antibody; genotype TR52, (**D**–**F**) anti-LigIV antibody, genotype B81, and (**G**–**I**) anti-γH2AX antibody; genotype B60. (**B**,**E**,**H**) Anti-rabbit Alexa Fluor^®^488-conjugated secondary antibody (green fluorescence) was used. (**I**) Ectopic association between chromosomes is indicated by an arrow (enlargement in the small box). Bar represents 10 µm.

Our data on the immunodetection of RAD51 (Figure 3A–C) and LigIV (Figure 3D,E) on condensed chromosomes suggest that DSB repair by HR and NHEJ is not limited to interphase, instead continuing during M-phase.

**Figure 4:**
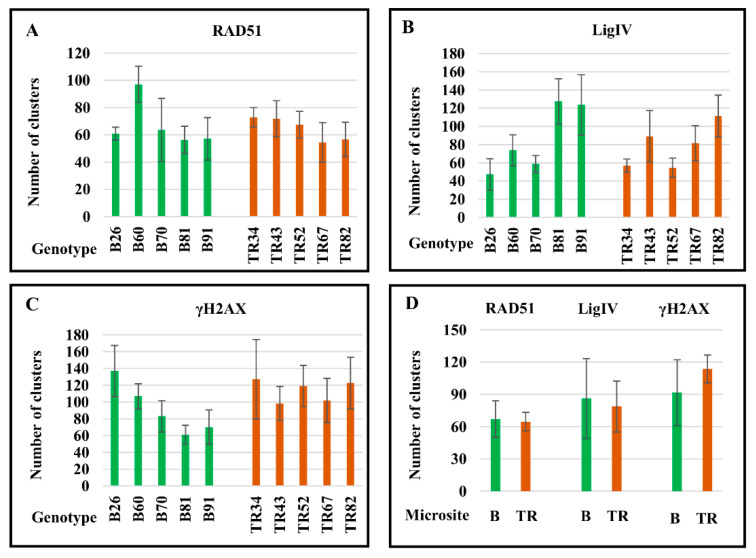
Number of clusters of RAD51 (**A**), LigIV (**B**), and γH2AX (**C**) on metaphase chromosomes in individual plants of the Basalt (B) and the Terra Rossa (TR) microsites of the Tabigha population. (**D**) The mean numbers of clusters are represented for the B and TR microsites. Standard error bars are shown.

At the same time, immunodetection of γH2AX evidences a large number of unresolved DSBs on mitotic chromosomes (Figure 3G–I). We analyzed 10 to 11 metaphase plates on each cytological slide, and significant variability in the number of RAD51, LigIV, and γH2AX foci between individual cells (Table S1) and between genotypes (Table 1, Figure 4) was documented.

For example, 2.7-fold differences in the number of RAD51 clusters were discovered between cells in genotype B70, 2.2-fold differences in the number of LigIV clusters were documented in the B26 genotype, and 2.6-fold differences were revealed in the number of γH2AX clusters between single cells in genotype TR34 (Table S1). Between genotypes, the biggest differences in the mean numbers of foci per metaphase plate were revealed for LigIV. The smallest number of clusters of LigIV per diploid chromosome plate was found in genotype B26, at 47, and the largest number of clusters was documented in genotype B81, at 127 (Table 1). Variability from 54 to 97 clusters was obtained for RAD51 between plants TR67 and B60, respectively (Table 1). Differences of 2.2 times were found between genotypes B81 and B26, which carry a minimum of 61 and a maximum of 137 γH2AX clusters, respectively (Table 1).

Thus, immunolocalization of RAD51 and LigIV on condensed metaphase chromosomes indicates post-interphase DSB repair by the HR and NHEJ mechanisms. In contrast, γH2AX foci on chromosomes indicate that much DNA damage remains unresolved in the M-phase of the cell cycle.

### 2.3. R-Loop Formation on Metaphase Chromosomes

**Figure 5:**
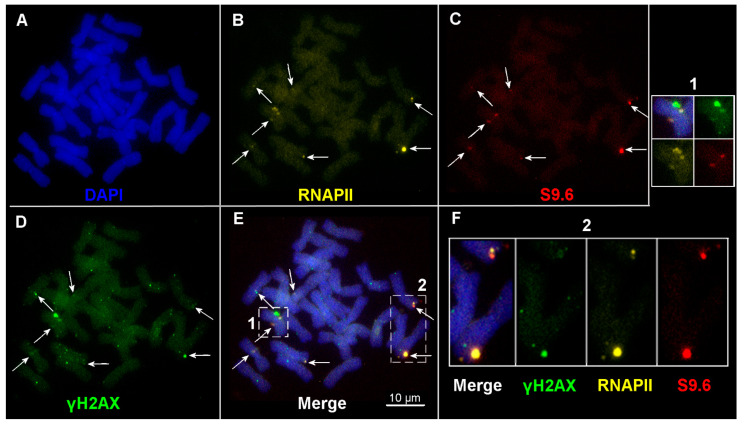
Immunodetection of clusters of RNAPII, S9.6, and γH2AX on metaphase chromosomes; genotype B81. (**A**) Counterstaining with DAPI. Immunofluorescent localization of (**B**) anti-RNAPII Alexa Fluor^®^546-conjugated antibody (in yellow), (**C**) anti-DNA-RNA hybrid [S9.6] FluoroProbes^®^647-conjugated (in red) antibody, (**D**) anti-γH2AX antibody; anti-rabbit Alexa Fluor^®^488-conjugated secondary antibody (in green). (**E**) Merged image. (**F**) The fragments in dashed boxes 1 and 2 in (**E**) are shown as enlargements in small boxes. The seven co-localized clusters of RNAPII, S9.6, and γH2AX are indicated by arrows. Bar represents 10 µm.

**Figure 6:**
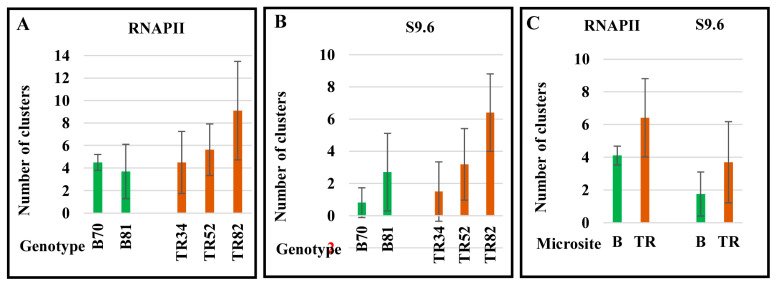
Number of clusters of RNAPII (**A**) and S9.6 (**B**) on metaphase chromosomes in individual plants of the Basalt (B) and the Terra Rossa (TR) microsites in the Tabigha population. (**C**) The mean numbers of clusters are represented for B and TR microsites. Standard error bars are shown.

Immunodetection of RNAPII and DNA-RNA hybrid [S9.6] indicates post-interphase R-loop formation on metaphase chromosomes (Figure 5). RNAPII and S9.6 chromosomal foci of different sizes and fluorescence intensities were revealed. Clusters of RNAPII and S9.6 can be adjacent to each other and co-localized with γH2AX foci on chromosomes, as shown in Figure 5. At the same time, there are single clusters of both RNAPII and S9.6; in most cases, the total number of RNAPII foci exceeded the number of S9.6 foci in single cells. The amounts of RNAPII and S9.6 clusters ranged from 0 to 18 and 0 to 10, respectively, between individual cells (Table S1, Figure 6). The greatest number of RNAPII and S9.6 clusters in a single cell was found in genotype TR82 (Figure 6; Table 1 and Table S1).

Individual genotypes differ significantly in the average number of RNAPII and S9.6 foci, and plants of the Terra Rossa carry higher amounts of RNAPII and S9.6 clusters compared with plants of the Basalt microsite (Figure 6; Table 1 and Table S1). Thus, in the M-phase of the cell cycle, active transcription sites marked with clusters of RNAPII and S9.6, R-loops primarily associated with γH2AX foci were detected on condensed chromosomes. The number of RNAPII and S9.6 foci in plants of the Terra Rossa site exceeded that of plants of the Basalt microsite.

### 2.4. The Number of DSBs and Transcription Sites in Interphase Nuclei in Plants of the Terra Rossa Microsite Is Higher Than in Plants of the Basalt Microsite

**Figure 7:**
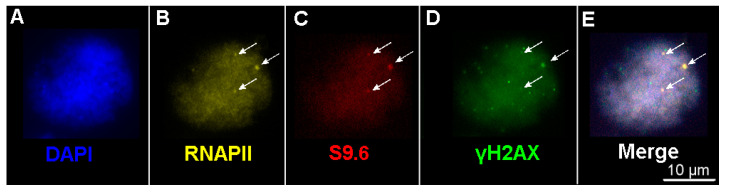
Immunodetection of clusters of RNAPII, S9.6, and γH2AX in interphase nuclei; genotype TR34. (**A**) Counterstaining with DAPI. Immunofluorescent localization of (**B**) anti-RNAPII Alexa Fluor^®^546-conjugated antibody (in yellow); (**C**) anti-DNA-RNA hybrid [S9.6] FluoroProbes^®^647-conjugated antibody (in red); (**D**) anti-γH2AX antibody; anti-rabbit Alexa Fluor^®^488-conjugated secondary antibody (in green). (**E**) Merged image. The co-localization of RNAPII, S9.6, and γH2AX large clusters is indicated with arrows. Bar represents 10 µm.

**Figure 8:**
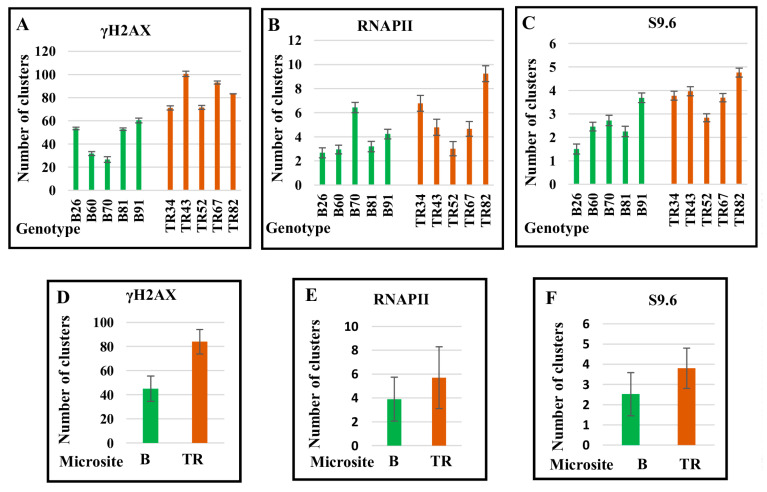
Number of clusters of γH2AX (**A**), RNAPII (**B**), and S9.6 (**C**) in interphase nuclei in individual plants of the Basalt (B) and the Terra Rossa (TR) microsites of the Tabigha population. Average number of clusters of γH2AX (**D**), RNAPII (**E**), and S9.6 (**F**) in the B and TR microsites. Standard error bars are represented.

Along with an intense dispersed signal, clusters of γH2AX, RNAPII, and S9.6 of different sizes and fluorescence intensities were revealed in interphase nuclei (Figure 7). Clusters small enough to be confused with the background were not included in the account (Figure 8, Table 2).

The number of γH2AX foci in interphase nuclei varied, on average, from 26.6 in genotype B70 to 100.6 in genotype TR43 (Table 2). The number of γH2AX foci in nuclei was 2 to 2.6 times lower than that on metaphase chromosomes in some plants (genotypes B26, B60, B70, TR34, and TR52), whereas in other genotypes (B81, B91, TR43, TR67, and TR82), these numbers were close in magnitude in both nuclei and chromosomes (Table 1 and Table 2). For example, in genotype TR67, on average, there were 81 clusters per metaphase plate and 100 clusters per nucleus, whereas for genotype B26, differences of 2.6 times were revealed in the number of γH2AX clusters between nuclei and chromosomes.

The average number of RNAPII and S9.6 clusters in the nuclei varied between plants. The greatest number of clusters, both in nuclei and chromosomes, was documented in genotype TR82 (Table 1 and Table 2). On average, the number of γH2AX, RNAPII, and S9.9 clusters in Terra Rossa plants exceeded that in plants at the Basalt microsite (Figure 8, Table 2).

Thus, the number of unresolved DSBs in interphase nuclei varies between genotypes, and plants of the Terra Rossa microsite have more DNA lesions than plants of the Basalt microsite. In addition, the number of transcription sites varies between individual plants and is larger in the Terra Rossa microsite.

## 3. Discussion

Our data indicate that the processes of DNA repair in the *T. dicoccoides* genome depends on the edaphic conditions in two adjacent native microsites. The Tabigha microsite consists of a transect of 100 m subdivided between hard Middle Eocene limestone weathering into dry and calcareous terra rossa red soil, abutting Pleistocene volcanic basalt flows, then weathering into humid and siliceous basalt soil [10]. In the Terra Rossa microsite, water percolation is high, and the upper soil layers dry out rapidly. In the experimental conditions, the amount of water added to the terra rossa pots to maintain them at constant weight was higher than that added to the controls [34]. The physicochemical differences drastically affect the structure and phenology of plant formations on the terra rossa and basalt soils in the Tabigha population [35]. On the terra rossa soil, *T. dicoccoides* plants (primarily the yellow spike morph) are lower in height and less abundant than on basalt (primarily the black spike morph). Plants growing on terra rossa terminate their life cycle and dry up in the spring, at most, 3–4 weeks after the last effective rains in contrast to the 8–10 weeks on basalt soil [35].

The DNA repair network combats the detrimental effects of exogenous and endogenous factors that affect genome integrity and genetic stability. In plants of the humid Basalt microsite, the average number of γH2AX foci indicating DSBs in the nuclei was almost two times less than that in plants of the Terra Rossa microsite (Figure 8, Table 2). Clearly, plants growing in the Terra Rossa microsite experience water deficiency; along with other factors, this causes an increase in the proportion of DNA damage during the cell cycle. Concurrently, the average level of expression of the DNA repair genes *MRE11* (DDR pathway), *LigIV* and *KU70* (NHEJ pathway), *MLH1* and *MSH2* (MMR pathway) in plants of the Terra Rossa microsite is lower compared with plants of the Basalt microsite (Figure 2), and differences between individual genotypes (Figure 1) in most cases are consistent with the average values. Therefore, it can be assumed that, on the one hand, the frequency of DSBs increases under drought stress, and on the other, a decrease in the expression level of genes involved in the DDR, NHEJ, and MMR pathways leads to a lower efficiency of DNA repair in plants growing in dry terra rossa soil compared with plants growing in humid basalt soil. However, the level of variability between individual genotypes, as well as the average values of *RAD51* (HR pathway) expression, were quite similar in the Basalt and Terra Rossa microsites (Figure 1 and Figure 2).

Unlike other genes, the mean expression of *BRCA1* is higher in the Terra Rossa microsite, and interindividual differences in the relative expression level are the greatest compared with those of other genes. Along with a key role in HR, *BRCA1* is involved in other interactions in eukaryotic cells, including NHEJ [36,37]. Clearly, drought also affects these processes in plants growing in the Terra Rossa microsite. Likewise, *MSH2* functions in at least two other complexes, MSH2-MSH3 and MSH2-MSH6, which have lesion recognition and DNA binding activities in the MMR pathway [21]. Moreover, the MMR and HR pathways can be mutually dependent, and MMR proteins are involved in HR [33,38,39]. Homoeologous genes of the MRN complex, including *MRE11*, are expressed at different levels in different polyploid wheat species and different tissues—specifically, roots, leaves, and meiocytes [40]. As a complement to this finding, our data indicate the influence of abiotic factors in the wild population on the expression level of *MRE11*.

Obviously, an adaptation of the *T. dicoccoides* genome to contrasting edaphic environments is associated with all cellular processes, as was recently shown for wild barley, *Hordeum spontaneum* K. Koch, which shares the Basalt and Terra Rossa microsites of the Tabigha population with wheat [41,42]. Edaphic adaptation of *H. spontaneum* to the basalt and terra rossa soils is regulated at epigenomic, transcriptomic, and metabolomic levels. Whole genome bisulfite sequencing, RNA-sequencing, and metabolome analysis reveal that the primary and secondary metabolisms are more active in Terra Rossa and Basalt barley plants, respectively. Some DNA methylation events are closely associated with the transcriptional levels of genes participating in fundamental biological processes. RNA-sequencing in leaves and roots revealed differences in transcriptome profiles between the barley plants from the Basalt and Terra Rossa microsites. In plants from the Terra Rossa, the upregulation of 712 and 567 differentially expressed genes (DEGs) and downregulation of 497 and 752 DEGs in comparison with the Basalt plants, respectively, were revealed. Adaptation of wild barley to contrasting edaphic environments appears to be mediated by key genes and metabolites associated with sugar metabolism in dry terra rossa soil and phentolamide biosynthesis in moist, fungi-rich basalt soil [42].

Environments directly affect the integrity and architecture of the plant genome that can be expressed in chromosomal aberrations, an indicator of both biotic and abiotic stresses. Thus, high polymorphism in chromosomal patterns of several types of repetitive DNA was documented in *T. dicoccoides* plants from two small Israeli populations growing in micro climatically contrasting microsites in “Evolution Canyon” (EC) [43]. In the EC, wild wheat inhabits two opposite slopes of Mount Carmel: a forested, wet, and cool north-facing temperate slope (NFS) and a tropical hot and dry savannoid south-facing slope (SFS). In the drought-stressed south-facing slope, two groups of plants, SFS1 and SFS2, were distinguished. In SFS1 plants, a series of homozygous translocations and the formation of tetravalent and anaphase bridges during meiosis were revealed. These chromosomal rearrangements caused hybrid sterility in crossing SFS1 and SFS2 plants [43].

To maintain genome stability, DNA repair systems must prevent chromosome aberrations and missegregation. The appearance of γH2AX foci on wheat chromosomes indicates that some proportion of DSBs remains unresolved during interphase. Moreover, in half of the plants, the number of γH2AX foci in metaphase chromosomes (Table 1) is greater than that in interphase nuclei (Table 2); this points to the occurrence of additional DNA damage during late mitosis. One of the reasons for the appearance of DSBs in the M-phase may be chromatin condensation. In particular, chromatin condensation leads to breaks in interchromosomal ectopic associations that arise in interphase as a result of illegitimate recombination and erroneous DNA repair. In the case of DSBs, ectopic sequences—especially tandem repeats and transposable elements (TEs)—may serve as the template for non-allelic DNA repair via template switching [44,45] in the somatic interphase [12,31,44,46,47], leading to ectopic chromosome associations found in the M-phase [48,49] (Figure 3I). Chromatin condensation toward metaphase and chromosome segregation in anaphase are accompanied by disruptions in illegitimate associations and DNA breaks. The appearance of RAD51 and LigIV foci on chromosomes indicates that the HR and NHEJ mechanisms combat late-occurring DNA damage; however, remaining unrepaired DSBs could provoke chromosomal aberrations in the next cell cycle. The smallest number of γH2AX foci was found on metaphase chromosomes of genotypes B81 and B91, whereas the number of LigIV clusters was two to three times higher than that in the eight other plants (Figure 4). At the same time, the number of RAD51 clusters varied only slightly between 10 plants, except for genotype B60. Likely, at the stage of condensed chromosomes, the efficiency of DSB repair is largely determined by NHEJ. In addition, the comparative patterns of γH2AX and LigIV—i.e., the trend toward a decrease in chromosomal DSBs with an increase in the number of LigIV clusters in all five Basalt plants and three Terra Rossa genotypes (TR34, TR43, and TR52)—also indicate a greater contribution of NHEJ compared with HR in DSB repair in condensed chromosomes.

DSB repair in interphase is achieved by cell cycle arrest at the G1/S and G2/M boundaries to resolve DNA breaks until the cell enters mitosis; specific mechanisms operate on the mitotic cell cycle checkpoint [50]. However, data are accumulating that confirm DNA repair occurring in the M-phase of mitosis. In cancer cells, Mitotic DNA repair Synthesis (MiDAS) induced by replication stress is associated with common fragile sites (CFSs), which are difficult-to-replicate loci manifesting as gaps or breaks on metaphase chromosomes [51]. The *RAD52*-dependent type of mitotic DNA repair synthesis, DDR-associated MiDAS, occurs at the sites of DSBs. Another type is non-DDR-associated MiDAS, which is *RAD51*-dependent and occurs irrespective of the induction of DNA damage [52]. The endonucleases MUS81/EME1 and BLM helicase are employed in CFSs and induce MiDAS, which minimizes chromosome aberrations and missegregation in the anaphase [51,53]. It was shown for human and rat kangaroo cells that micro-focused laser-induced DNA damage in mitotic chromosomes undergoes DNA repair synthesis; mitotic DNA-damaged cells enter subsequent interphase, and DNA repair continues in the G1 phase [54,55]. DSBs on metaphase chromosomes lead to the clustering of all three components of the MRN complex and proteins involved in NHEJ and HR, suggesting the recruitment of different DNA repair pathways in mitosis [54,55,56]. Thus, similar to the mammalian genomes, RAD51 and LigIV foci on condensed chromosomes indicate ongoing DSB repair via the HR and NHEJ pathways during the M-phase in wheat.

Our data evidence that R-loop formation on condensed chromosomes in the M-phase (Figure 5) is another potential source of chromosomal aberrations and genome instability. Generation of R-loops at transcriptionally active sites is a naturally occurring process in eukaryotic cells, including plants [14,15,17,57]. R-loops play an important role in gene expression, replication, and chromatin structure, whereas accumulation of aberrant R-loops causes DNA damage, particularly DSBs; in turn, DNA damage can initiate R-loop formation [14,58,59]. Adjacent S9.6 and RNAPII foci are co-localized with γH2AX clusters and are also detected as single foci on wheat chromosomes (Figure 5). It can be assumed that both scenarios are realized in the M-phase in the wheat genome. In other words, unresolved DSBs can initiate R-loop formation, while R-loops can induce DSBs at the end of mitosis.

Repetitive DNA sequences are hotspots of DNA damage and chromosomal aberrations, and R-loops accumulate in regions of satellite repeats and transposable elements—particularly in ribosomal DNA, centromeres, and telomeres—in various species, including plants [57,60,61,62]. Moreover, R-loops can be generated from TEs [57,63]. The large genomes of polyploid wheats and their diploid relatives are highly enriched in various types of repetitive DNA sequences, primarily tandem repeats and TEs [64,65], which exhibit a pan-chromosomal dispersal distribution, as well as chromosome-, species-, and sequence type-specific cluster patterning [47,66,67,68]. It is reasonable to assume that large chromosomal foci of RNAPII and S9.6 on metaphase chromosomes are mainly associated with clusters of repetitive sequences, particularly TEs, which are hotspots of DNA damage and chromosomal aberrations. Evidently, the increase in the number of DSBs and R-loops in plants of the Terra Rossa microsite compared with plants of the Basalt microsite is associated with the effect of edaphic factors on repetitive DNA fractions of the wheat genome.

Overall, our results show that *T. dicoccoides* genome plasticity and adaptability to contrasting environments imply the mobilization of various DNA repair mechanisms in varying proportions to maintain genome integrity and generate genetic diversity.

## 4. Materials and Methods

### 4.1. Plant Material

Native *T. dicoccoides* plants growing on two microsites, Basalt and Terra Rossa, in the population of Tabigha (Institute of Evolution collection, Israel) were analyzed using the IF and real-time qPCR approaches. Five plants from each microsite were taken for analysis. The genotype abbreviations are as follows: plants collected in the Terra Rossa (TR) microsite: genotypes TR34, TR43, TR52, TR67, and TR82; plants collected in the Basalt (B) microsite: genotypes B26, B60, B70, B81, and B91.

The population of Tabigha is located north of the Kinneret Lake in Upper Galilee as low as 100 m below sea level (Latitude 32.8982, Longitude 35.5493 Rosensaft, M., Sneh, A., 2020. Geological map of Israel, Sheet:Teverya (2008). GSIWebApp #64/2020, GISunit, Geological Survey of Israel, 2020). Topographically, the shallower terra rossa is hillier than the flat, deeper basalt soil. In terra rossa, the terrain is dominated by numerous bare rocks interspersed with pockets of soil [35]. The total available phosphorus is much higher in terra rossa than in other soil types [34]. The terra rossa soil niche turned out to be clearly drier than the basalt niche in terms of water and moisture availability throughout the growing season (November to May) [35]. The percentage of available soil moisture (field capacity-wilting point) in terra rossa is almost half that of other soil types in the region, including basalt [34].

### 4.2. Total RNA Isolation and Reverse Transcription

Total RNA was isolated from five plants collected in the Basalt microsite and five plants collected in the Terra Rossa microsite of the Tabigha population. RNA extraction was performed from young seedlings at the stage of five to seven leaves. The two youngest leaves (100 mg) were immediately frozen in liquid nitrogen and ground to a fine powder with a mortar and pestle. Total RNA was isolated using a NucleoSpin^®^ RNA Plant kit (MACHEREY-NAGEL GmbH & Co. KG, Germany) according to the manufacturer’s instructions. The quality and quantity of RNA samples were assessed spectrophotometrically using a NanoDrop^TM^ Spectrophotometer 2000 (Thermo Fisher Scientific, Waltham, MA, USA). The RNA from all the samples was of similar purity and quality. Reverse-transcription PCR was performed to generate cDNA with an iScript™ cDNA Synthesis Kit (Bio-Rad, Hercules, CA, USA) following the manufacturer’s instructions.

### 4.3. Real-Time Quantitative PCR and Primer Design

Primers for qPCR were designed using Primer Express 2.0 software (Table S2). The reactions were performed in a 20 µL reaction volume containing 10 µL of 2X Fast SYBR™ Green Master Mix (Applied Biosystems, San Francisco, CA, USA), 0.75 µL of the forward primer (10 µmol), 0.75 µL (10 µmol) of the reverse primer, 5 ng of cDNA as the template, and 3.5 µL of ultra-pure water. The qPCR reactions were analyzed using a StepOne™ Plus Real-Time PCR system and StepOne Software version 2.2.2 (Applied Biosystems). The following reaction parameters were used: 20 s at 95 °C, followed by 40 cycles at 95 °C for 3 s, and 60 °C for 30 s. The PCR product melting curves were assessed to verify that there was a single product for each primer pair, with a range of 60–95 °C and a step of 0.3 °C. In addition, single qPCR products were validated using electrophoresis in 1.5% agarose gels. The efficacy of the PCR reactions was confirmed based on a correlation coefficient value of ~1 and a slope of −3.3 ± 0.1 of the standard curve generated by a serial 10-fold dilution of the cDNA of the sample that was assigned as “1”. The reactions were performed in triplicate. In the control wells containing ultrapure double-distilled water (instead of template DNA), no amplification was detected. The relative expression (RE) level of genes was evaluated based on the ∆∆Ct method using the wheat Actin gene as the reference gene (∆Ct_Target_ = Ct_Target_ − Ct_,Actin_; ∆∆Ct = ∆Ct_Target_ − ∆Ct_Const_; RE = 2^−∆∆Ct^) [69].

### 4.4. Preparation of Cytological Slides

Cytological slides of individual shoot meristems containing interphase nuclei and well-spread chromosomal plates were used. Chromosome spreading was conducted as previously described [70,71], with some modifications. Seeds were germinated on moist filter paper at 24 °C in the dark. Seedlings of 5–7 mm in length were transferred to ice water for 24–26 h to accumulate metaphases and then fixed in 3:1 (*v*/*v*) 100% ethanol:acetic acid for 5 days or longer. Tissues were then washed (3 × 5 min) in water and incubated in an enzyme buffer (10 mM citrate buffer at pH 6.0) and partially digested for 1 h at 37 °C in 10% pectinase plus 0.5% cellulase (NBC Biomedicals, High Wycombe, UK) plus 5% “Onozuka” R-10 cellulase (Yakult Honsha Co., Ltd., Tokyo, Japan), followed by washing in enzyme buffer (3 min) and distilled water (2 × 5 min). The shoot meristem, in a drop of water, was carefully transferred onto a grease-free microscope slide using metal stainless steel needles; the cells were spread in the drop of 60% acetic acid at 45–47 °C for 40 to 50 s, fixed in 3:1 (*v*/*v*) 100% ethanol:acetic acid and immersed in absolute ethanol for 3–5 s. Dry chromosome spreads without cytoplasm was used for IF.

### 4.5. Fluorescence Immunolocalization

Slides were immersed in 1% phosphate-buffered saline (PBS) buffer containing 0.1% Triton for 3 × 5 min. They were then incubated in a blocking buffer (1% PBS + 0.1% Triton + 1% bovine serum albumin [BSA]) for 1 h at room temperature. The primary antibody, diluted in blocking buffer, was applied to the slides, and the slides were incubated overnight at 4 °C in a moisture chamber. The following primary antibodies were used: rabbit polyclonal anti-γH2A.X (phospho S139) antibody (#ab2893, Abcam, Cambridge, UK; 1:1000 dilution), custom rabbit polyclonal anti-*T. aestivum* (wheat) DNA Ligase IV antibody (#DZ41283, BOSTER, Pleasanton, CA, USA; 1:1000 dilution), rabbit polyclonal anti-RAD51 antibody (#A6268, ABclonal, Woburn, MA, USA; 1:1000 dilution), mouse monoclonal anti-Pol II antibody (Pol II (8WG16) Alexa Fluor^®^546 conjugated (#SC-56767 AF546, Santa Cruz Biotechnology, Inc., USA; 1:100 dilution), goat anti-DNA-RNA hybrid [S9.6] FluoroProbes^®^647 conjugated antibody (#Ab01137-24.1, Absolute Antibodies, Oxford, UK; 1:50 dilution). The slides were then washed (2 × 5 min) in 1% PBS + 0.1% Triton buffer, and a secondary antibody, donkey anti-rabbit Alexa Fluor^®^488 (#ab150073, Abcam) diluted in blocking buffer (1:1000), was applied. The slides were then incubated for 1 h at room temperature. Following this, the slides were washed in 1% PBS + 0.1% Triton buffer (3 × 5 min), counterstained with 2.5 µg/mL DAPI for 10 min, and mounted in VECTASHIELD antifade mounting medium (Vector Laboratories, Newark, CA, USA).

### 4.6. Microscopy and Image Analysis

The slides were examined on a Leica DMi8 fluorescent inverted microscope with an HC PL APO 100×/1.40–0.70 oil objective using Leica Application Suite X (LAS X) software v.4.70 (Leica Microsystems, Wetzlar, Germany). Confocal imaging was done using a Nikon A1R Confocal Laser Scanning Inverted Microscope with a Plan Apo 63×/1.4 oil objective using NIS Elements C-ER software v.4.50 (Nikon Instruments Inc., Mel-ville, NY, USA). Images of z-stacks of 15–20 slices × 0.4 µm per specimen were acquired. The lasers and filters used were as follows: 406 nm, filter 450/50 for DAPI; 492 nm, filter 525/50 for AF488; 561 nm, filter 595/50 for Cy3; and 639 nm, filter 700/75 for AF647.

For three-dimensional (3D) reconstruction modeling and analysis, images of interphase nuclei with γH2AX, RNAPII, and S9.6 foci were processed using the Imaris Surface reconstruction module and the Spots Module (Bitplane). RAD51, LigIV, RNAPII, and S9.6 foci on chromosomes were counted manually.

## Figures and Tables

**Table 1:** Mean number of RAD51, LigIV, γH2AX, RNAPII, and S9.6 clusters on metaphase chromosomes in plants of the Basalt (B) and the Terra Rossa (TR) microsites of the Tabigha population. For each genotype, 10 to 11 metaphase plates were analyzed (see Table S1).

	Mean Number of Clusters per Chromosome Plate (SE)				
	RAD51	LigIV	γH2AX	RNAPII	S9.6
	**Basalt microsite**				
**Genotype**					
B26	60.9 (±4.8)	47.3 (±17.3)	136.9 (±30.5)	NC	NC *
B60	97.0 (±13.3)	73.8(±16.9)	106.0 (±14.9)	NC	NC
B70	63.5 (±18.8)	58.6(±9.3)	83.1 (±0.7)	4.5 (±0.7)	0.8 (±0.9)
B81	56.3 (±10.1)	127.4(±24.8)	61.1 (±11.1)	3.7 (±2.4)	2.7 (±2.4)
B91	57.1 (±15.5)	123.6 (±33.2)	70.1 (±20.6)	NC	NC
average	67.0 (±17.0)	86.1 (±37.2)	91.6 (±30.6)	4.1 (±0.6)	1.8 (±1.3)
	**Terra Rossa microsite**				
**Genotype**					
TR34	72.9 (±7.1)	56.8 (±7.2)	127.1 (±47.2)	4.5 (±2.8)	1.5 (±1.8)
TR43	71.8 (±13.2)	89.1 (±28.4)	98.2 (±20.3)	NC	NC
TR52	67.4 (±9.8)	54.5 (±10.6	119.0 (±24.6)	5.6 (±2.3)	3.2 (±2.2)
TR67	54.4 (±14.6)	81.5 (±19.4)	101.8 (±26.2)	NC	NC
TR82	56.6 (±12.6)	111.3 (±22.9)	122.7 (±30.7)	9.1 (±4.4)	6.4 (±2.4)
average	64.6 (±8.6)	78.7 (±23.7)	113.8 (±12.9)	6.4 (±2.4)	3.7 (±2.5)

* NC–Non-checked.

**Table 2:** Mean number of γH2AX, RNAPII, and S9.6 clusters in interphase nuclei in plants of the Basalt (B) and Terra Rossa (TR) microsites of the Tabigha population.

	Number of Nuclei Analyzed	Mean Number of Clusters per Nucleus (SE)		
		γH2AX	RNAPII	S9.6
**Basalt microsite**				
**Genotype**				
B26	56.0	53.4	2.7	1.5
B60	72.0	31.7	2.9	2.5
B70	53.0	26.6	6.4	2.7
B81	52.0	52.8	3.2	2.3
B91	60.0	60.3	4.2	3.7
average		45.0 (±10.4)	3.9 (±1.8)	2.5 (±1.1)
**Terra Rossa microsite**				
**Genotype**				
TR34	53.0	71.2	6.8	3.8
TR43	52.0	100.6	4.8	4.0
TR52	67.0	71.6	3.0	2.8
TR67	61.0	93.1	4.7	3.7
TR82	54.0	83.4	9.2	4.8
average		84.0 (±10.2)	5.7 (±2.6)	3.8 (±1.0)

## Data Availability

Not applicable.

## Supplementary Materials

The following supporting information can be downloaded at: https://www.mdpi.com/article/10.3390/ijms24076847/s1.

## Abbreviations

*BRCA1*	Breast cancer susceptibility gene 1
DAPI	4′,6-diamidino-2-phenylindole
DDR	DNA Damage Response
HR	Homologous Recombination
*KU70*	X-ray cross complementing 6
*LigIV*	DNA ligase 4
*MLH1*	MutL homolog 1
MMR	Mismatch Repair
*MRE11*	Meiotic Recombination 11
*MSH2*	MutS homolog 2
NHEJ	Non-Homologous End Joining
*RAD51*	Radiation sensitive 51
RNAPII	RNA polymerase II
γH2AX	gamma-H2AX, phosphorylated H2AX
