# Supplementary material for: The Influence of Edaphic Factors on DNA Damage and Repair in Wild Wheat Triticum dicoccoides Körn. (Poaceae, Triticeae)
Source: Int J Mol Sci. 2023 Apr 6;24(7):6847. doi: 10.3390/ijms24076847 (PMC10094829; doi:10.3390/ijms24076847)
Supplement: Supplementary file 1 [file ijms-24-06847-s001.zip › ijms-2269409-supplementary.pdf]

Table S1. Numbers of clusters of RAD51, LigIV, γH2AX, RNAPII, and S9.6 detected on single metaphase chromosome plates in individual plants.

* RNAPII and S9.6 clusters were counted together with γH2AX clusters on the same cytological slide.
**NC – Non-checked.

		**Number of clusters per single chromosome plate**				
**Genotype**	**Plate No.**	**RAD51**	**LigIV**	**γH2AX**	**RNAPII ***	**S9.6 ***
**B26**	1	65	37	127	NC	NC**
	2	62	43	149	NC	NC
	3	54	26	184	NC	NC
	4	60	76	185	NC	NC
	5	69	40	140	NC	NC
	6	57	44	109	NC	NC
	7	67	36	101	NC	NC
	8	58	75	124	NC	NC
	9	59	61	148	NC	NC
	10	58	35	102	NC	NC
**average**		**60.9**	**47.3**	**136.9**	NC	NC
**SE**		±4.8	±17.3	±30.5	NC	NC
**B60**	1	85	99	115	NC	NC
	2	115	86	102	NC	NC
	3	99	50	103	NC	NC
	4	112	60	90	NC	NC
	5	88	69	93	NC	NC
	6	92	55	110	NC	NC
	7	114	81	124	NC	NC
	8	98	62	117	NC	NC
	9	75	82	85	NC	NC
	10	92	56	130	NC	NC
	11		94		NC	NC
**average**		**97.0**	**72.2**	**106.9**		
**SE**		±13.3	±16.9	±14.9		
**B70**	1	58	51	63	5	2
	2	59	55	64	4	0
	3	36	48	68	4	0
	4	42	52	75	4	0
	5	96	66	83	5	1
	6	92	56	84	4	2
	7	37	63	85	5	0
	8	95	70	86	6	0
	9	59	75	96	4	1
	10	61	50	127	4	2
**average**		**63.5**	**58.6**	**83.1**	**4.5**	**0.8**
**SE**		±23.2	±9.3	±18.8	±0.7	±0.9
**B81**	1	73	108	73	1	1
	2	45	109	73	2	2

	3	44	116	51	3	1
	4	69	108	48	5	4
	5	53	110	60	4	3
	6	64	128	50	7	5
	7	51	148	57	4	2
	8	61	187	71	8	8
	9	55	126	76	2	0
	10	48	134	52	1	1
**average**		**56.3**	**127.4**	**61.1**	**3.7**	**2.7**
**SE**		±10.1	±24.8	±11.1	±2.4	±2.3
**B91**	1	78	85	72	NC	NC
	2	40	96	69	NC	NC
	3	46	82	107	NC	NC
	4	45	105	58	NC	NC
	5	74	100	82	NC	NC
	6	70	134	82	NC	NC
	7	34	159	89	NC	NC
	8	69	163	49	NC	NC
	9	53	156	54	NC	NC
	10	62	156	39	NC	NC
**average**		**57.1**	**123.6**	**70.1**		
**SE**		±15.5	±33.2	±20.6		
**TR34**	1	61	69	146	6	4
	2	73	53	164	7	0
	3	68	57	97	7	0
	4	84	57	113	6	0
	5	76	63	97	0	5
	6	82	65	94	2	0
	7	75	49	123	5	2
	8	77	61	96	0	2
	9	72	47	98	6	0
	10	71	48	243	6	2
	11	63	56			
**average**		**72.9**	**56.8**	**127.1**	**4.5**	**1.5**
**SE**		±7.1	±7.2	±47.2	±2.8	±1.8
**TR43**	1	58	89	114	NC	NC
	2	64	59	74	NC	NC
	3	62	77	69	NC	NC
	4	58	82	87	NC	NC
	5	59	64	77	NC	NC
	6	90	56	110	NC	NC
	7	87	87	131	NC	NC
	8	88	124	106	NC	NC
	9	77	141	105	NC	NC
	10	75	112	109	NC	NC
**average**		**71.8**	**89.1**	**98.2**		

**SE**		±13.2	±28.4	±20.3		
**TR52**	1	84	78	79	7	5
	2	63	36	105	7	6
	3	69	57	107	3	2
	4	69	53	108	6	1
	5	70	52	110	2	4
	6	53	53	115	7	3
	7	64	52	118	7	7
	8	55	62	129	5	0
	9	81	49	125	2	1
	10	66	53	135	8	2
	11			178	8	4
**average**		**67.4**	**54.5**	**119.0**	**5.6**	**3.2**
**SE**		±9.8	±10.6	±24.6	±2.3	±2.2
**TR67**	1	38	61	81	NC	NC
	2	56	49	60	NC	NC
	3	52	76	131	NC	NC
	4	60	67	88	NC	NC
	5	28	86	99	NC	NC
	6	46	82	109	NC	NC
	7	75	95	86	NC	NC
	8	52	116	152	NC	NC
	9	71	86	99	NC	NC
	10	66	97	113	NC	NC
**average**		**54.4**	**81.5**	**101.8**		
**SE**		±14.6	±19.4	±26.2		
**TR82**	1	84	104	90	12	6
	2	40	136	100	8	10
	3	64	99	106	6	6
	4	60	79	198	15	8
	5	60	92	143	6	5
	6	40	138	106	8	8
	7	50	127	118	6	2
	8	57	91	134	18	6
	9	57	136	114	6	9
	10	54	111	118	6	4
**average**		**56.6**	**111.3**	**122.7**	**9.1**	**6.4**
**SE**		±12.6	±22.9	±30.7	±4.4	±2.4

Table S2. Primer combinations for real-time quantitative PCR.

No.		Sequence	NCBI Reference Sequence
1.	**Actin-F**	AGATGATGCACCAAGGGCTG	XM_037590498.1
2.	**Actin-R**	CCCCATCCCTACCATGACAC	
3.	**MRE11-F**	GGACGCAGCGAACGAAGTAA	XM_037623921.1
4.	**MRE11-R**	TTTCCGTCCACTTTGTGGGA	
5.	**KU70-F**	GATTGGGCAGACCTTGCAGA	XM_037583509.1
6.	**KU70-R**	GCGCCGACAGGTAAGTTTTC	
7.	**LigIV-F**	ACAAAGATCTGGCCGACGAG	XM_037631088.1
8.	**LigIV-R**	CATACATGCTACCCCGAGCC	
9.	**RAD51-F**	CGAGCACCTCCAGGCATCT	XM_037612996.1
10.	**RAD51-R**	CTCCACTGTGCAAAGACCAGC	
11.	**BRCA1-F**	AAATGTGCTTGCTGCGGAGT	XM_037565827.1
12.	**BRCA1-R**	TGAAGTGGAAGCTTTTGCGAC	
13.	**MSH2-F**	TGCATACAGCCACTGATCTGG	XM_037548313.1
14.	**MSH2-R**	AACACATGTCCAAATGGGCC	
15.	**MLH1-F**	ACACCCCTGATATGGATCGC	XM_037603461.1
16.	**MLH1-R**	TCTTTCTCAACGTCCCAGGC	
